# Relationship of masked obesity to self-reported lifestyle habits, ideal body image, and anthropometric measures in Japanese university students: A cross-sectional study

**DOI:** 10.1371/journal.pone.0281599

**Published:** 2023-02-21

**Authors:** Kaito Yamashiro, Naho Yamaguchi, Kazunori Sagawa, Shigeharu Tanei, Fumihiko Ogata, Takehiro Nakamura, Naohito Kawasaki

**Affiliations:** 1 Laboratory of Public Health, Faculty of Pharmacy, Kindai University, Higashi-Osaka, Osaka, Japan; 2 Antiaging Center, Kindai University, Higashi-Osaka, Osaka, Japan; 3 Faculty of Pharmaceutical Sciences, Nihon Pharmaceutical University, Kitaadachi-gun, Saitama, Japan; University/College Library, ETHIOPIA

## Abstract

**Introduction:**

Masked obesity (MO) is defined as a normal body mass index (BMI) with a high body fat percentage (%BF), and is associated with the onset of lifestyle-related diseases. However, little is known about the current status of MO. Therefore, we investigated the relationship of MO to physical characteristics and lifestyle habits among Japanese university students.

**Methods:**

Between 2011 and 2019, we conducted a survey of 10,168 males and 4,954 females with BMI within the normal range (18.5 ≤ BMI < 25 kg/m^2^). MO was defined as %BF ≥ 20% in males and %BF ≥ 30% in females. Students completed a questionnaire containing items about lifestyle habits. Systolic and diastolic blood pressures were measured, and hypertension was defined as systolic above 140 mmHg and/or diastolic blood pressure above 90 mmHg. A multivariate logistic regression analysis was performed to evaluate the relationships as follows: the relationship of masked obesity to self-reported lifestyle habits, ideal body image, and anthropometric measures; the relationship between hypertension and body indices.

**Results:**

The proportion of students with MO in 2019 was 13.4% in males and 25.8% in females, and the proportion of females increased over time. MO was associated with desire for weight loss (odds ratio, 95% confidence interval: 1.76, 1.53–2.02), intake of five macronutrients (0.79, 0.67–0.93), rice and wheat intakes (1.22, 1.01–1.47), sleep duration of < 7hr (0.85, 0.74–0.98), and exercise habit (0.71, 0.63–0.81) in males; and with balanced diet intake (0.79, 0.64–0.99) and exercise habit (0.65, 0.51–0.82) in females. There was a significant association of MO with hypertension in males (1.29, 1.09–1.53).

**Conclusion:**

The percentage of female students with MO increased during the study period, and in males, MO may be a risk factor for hypertension. These results suggest that intervention for MO is needed in Japanese university students.

## Introduction

Masked obesity (MO) is defined as normal body mass index (18.5 ≤ BMI < 25 kg/m^2^) but with a high body fat percentage (%BF). Various definitions of MO have been proposed in previous studies [1–6]. Fujise et al. [2] defined MO as a normal BMI with %BF of ≥ 20% in males and ≥ 30% in females. In a study of 8,068 young females [1], the proportion of participants with BMI ≤ 25 kg/m^2^ and %BF ≥ 30% was 7.4% of the total study population. Similarly, the proportion of young females with normal BMI and with %BF ≥ 30% has been reported as 32.0% [4]. Oguri et al. [5] reported that total cholesterol and triglyceride levels were significantly higher in those with MO. MO could induce lifestyle-related diseases such as hypertension, and the relationship should be investigated.

BMI is used to determine obesity; however, it is not a precise measure of body fat and thus may not accurately reflect the amount of body fat in an individual. New anthropometric indices that can estimate body fat distribution have recently been suggested for evaluation of obesity, including the body roundness index (BRI), body shape index (ABSI), body adiposity index (BAI), abdominal volume index (AVI), and conicity index (CI) [7, 8]. As these anthropometric indices can be a useful predictor of MO, it is necessary to investigate the relationship between them and MO; however, there are limited data on these relationships among Japanese university students.

It has been reported that young Japanese females with MO tended to have no exercise habit, more dieting (restricting calorie intake) experience, and lower intakes of vegetables and beans [4]. Similarly, MO groups were reported to consume fewer vegetables per day and consumed mayonnaise and fried foods more frequently compared with groups of normal body type [9]. Furthermore, poor eating behaviors that are caused by a desire for weight loss may induce MO [2]. Females are known to have a stronger desire for weight loss than males, and numerous studies have investigated the desire for weight loss and weight loss experienced by female university students [10–13]. Additionally, most previous studies [2, 4, 9] have focused on MO among female students, and corresponding data on male students is lacking. Thus, there is a need to investigate the relationship of MO to lifestyle habits and the desire for weight loss among both male and female Japanese students.

University students are known to have more flexible daily schedules compared with high school students and adults, as well as more inappropriate eating habits [14] and sleeping habits [15]. Physical characteristics that are apparent in young adulthood are not temporary, and are likely to be associated with those in middle age [16]. Additionally, MO could induce lifestyle-related disease such as hypertension, and it is desirable to improve inappropriate lifestyle habits during the early stage of life. Therefore, this study aimed to investigate the relationship of MO to physical characteristics and lifestyle, and evaluate its impact on the blood pressure of Japanese university students.

## Materials and methods

### Participants and survey period

The survey population consisted of students who were enrolled at Kindai University between 2011 and 2019. The number of students in the first grade is approximately 5,000 males and 2,500 females every year. Subjects for whom data on sex, age, lifestyle habits, blood pressure, and anthropometric measures were missing or unclear were excluded. Finally, 10,168 males and 4,954 females with BMI within the normal range (18.5 ≤ BMI < 25 kg/m^2^) were eligible for participation in the study. MO was defined as %BF ≥ 20% in males and ≥ 30% in females [2]. Although Fujise et al. [2] defined the normal range of BMI as 19.8 to 24.2 kg/m^2^, our study used the current standards of the Japan Society for the Study of Obesity (JASSO) [17]. In the total eligible population, MO was identified in 1,868 male and 915 female students (MO group). Those without MO were included in the normal group.

### Survey content and methodology

S1 Table lists questionnaire items related to age; sex; the desire for weight loss; and eating, sleeping, and exercise habits. The survey was conducted using self-administered questionnaires that were distributed, completed, and then collected during a university lecture. In terms of ideal body weight, students who answered that they “want to lose weight” were classified as the “decreasing group,” those who answered that they “want to maintain the same weight” were defined as the “maintenance group,” and those who answered that they “want to gain weight” were defined as the “increasing group.” Regarding snack intake, the respondents were asked to choose between “Daily,” “Occasionally,” “I used to snack,” or “I do not snack.” Those who answered “I do not snack” or “I used to snack” were classified as the “no-snacking group,” and those who answered with another option were classified as the “snacking group.” The respondents were asked to self-evaluate their intake of nutrients on a 10-point scale (1: dissatisfied to 10: satisfied). The items presented were five macronutrients, rice/wheat, meat, fat, vegetables, fruit, and the levels of their balance. Each item was dichotomized as the “low-value group” (≤ 5 points) and “high-value group” (≥ 6 points). Sleep duration was categorized as < 7 hours, 7 ≤, < 8 hours, or ≥ 8 hours; and sleep onset time as ≥ 15 minutes and < 15 minutes. The median sleep onset time was 15 minutes in male and female students in this study. In addition, exercise habit was defined based on the National Health and Nutrition Survey [18] as “exercise frequency” of at least twice a week, and as “exercise duration” of at least 30 minutes per exercise session.

### Blood pressure, anthropometric measures, and body indices

Systolic and diastolic blood pressures (SBP and DBP, respectively) were measured at the level of the heart after 5 minutes rest in the sitting position, using an automatic electronic sphygmomanometer (HEM-1010; Omron Dalian Co., Ltd., Kyoto, Japan). Hypertension was defined according to The Japanese Society of Hypertension (JSH) guidelines [19] as SBP > 140 mmHg and/or DBP > 90 mmHg. At the same time as blood pressure measurements were obtained, anthropometric parameters of height, weight, waist circumference (WC), and hip circumference (HC) were measured to the nearest 0.1 cm or 0.1 kg in the standing position, with the participants wearing minimal clothing. The %BF was measured using a dual-frequency body composition meter (DC-320 or DC-430A; Tanita Co., Tokyo, Japan). WC was measured at the level of the umbilicus, and HC was measured at the widest part around the buttocks. BMI, WC to HC ratio (WHR), and WC to height ratio (WHtR) were calculated using the following formulae. The body indices of ABSI [20], AVI [21], BAI [22], BRI [23], and CI [24] were calculated using the following formulae.


BMI=WeightkgHeightm2 (1)

WHR=WCcmHCcm (2)

WHtR=WCcmHeightcm (3)

ABSI=WCmHeightm12×BMIkgm223 (4)

AVI=2×WCcm2+0.7×WCcm-HCcm21000 (5)

BAI=HCcmHeightm1.5-18 (6)

BRI=364.2-365.5×1-WCm2π20.5×Heightm2 (7)

CI=WCm0.109×WeightkgHeightm (8)

### Statistical analysis

The Cochran–Armitage trend test was used to evaluate the proportion of MO in males and females over time. The Mann–Whitney U test was performed to compare anthropometric indices between the MO and normal groups. Receiver-operating characteristic (ROC) curve analysis was conducted to assess the predictive performance of the anthropometric indices for MO by calculating the area under the curve (AUC). The sensitivity and specificity of each anthropometric index were also determined using ROC analysis. The maximum value of Youden’s index (sensitivity + specificity − 1) was considered the optimal cut-off point for each anthropometric index, and the students were classified into high and low groups based on each cut-off point. Furthermore, a multivariate logistic regression analysis was performed to consider confounding factors. The objective variable was set to MO, and the explanatory variables were set to ideal body weight (decreasing or increasing vs. maintenance), eating habits, sleep duration (< 7 hours or ≥ 8 hours vs. 7 ≤, < 8 hours), sleep onset time, and exercise habits. Additionally, a multivariate logistic regression analysis was performed to evaluate the relationship between hypertension and body indices. The objective variable was set to hypertension, and the explanatory variables were set to BMI, WC, and MO in males, and WC in females. A forward and backward stepwise selection with a significance level of 0.05 was performed to evaluate the effectiveness of the explanatory variables. Windows JMP Pro ver. 15.00 (SAS Institute Inc., Cary, NC, USA) was used for statistical processing and significance tests, with a significance level of less than 5%.

### Ethical considerations

This study protocol was approved by the Research Ethics Committee of Kindai University (approved ID: 08–001, 12–033, and 15–072) and all participants provided written informed consent. The participants were informed that their data would only be used for research on antiaging or prevention of lifestyle-related diseases, that participation in this survey was voluntary, and that participants’ privacy would be guaranteed. There were no participants younger than 18 years of age.

## Results

**Fig 1:**
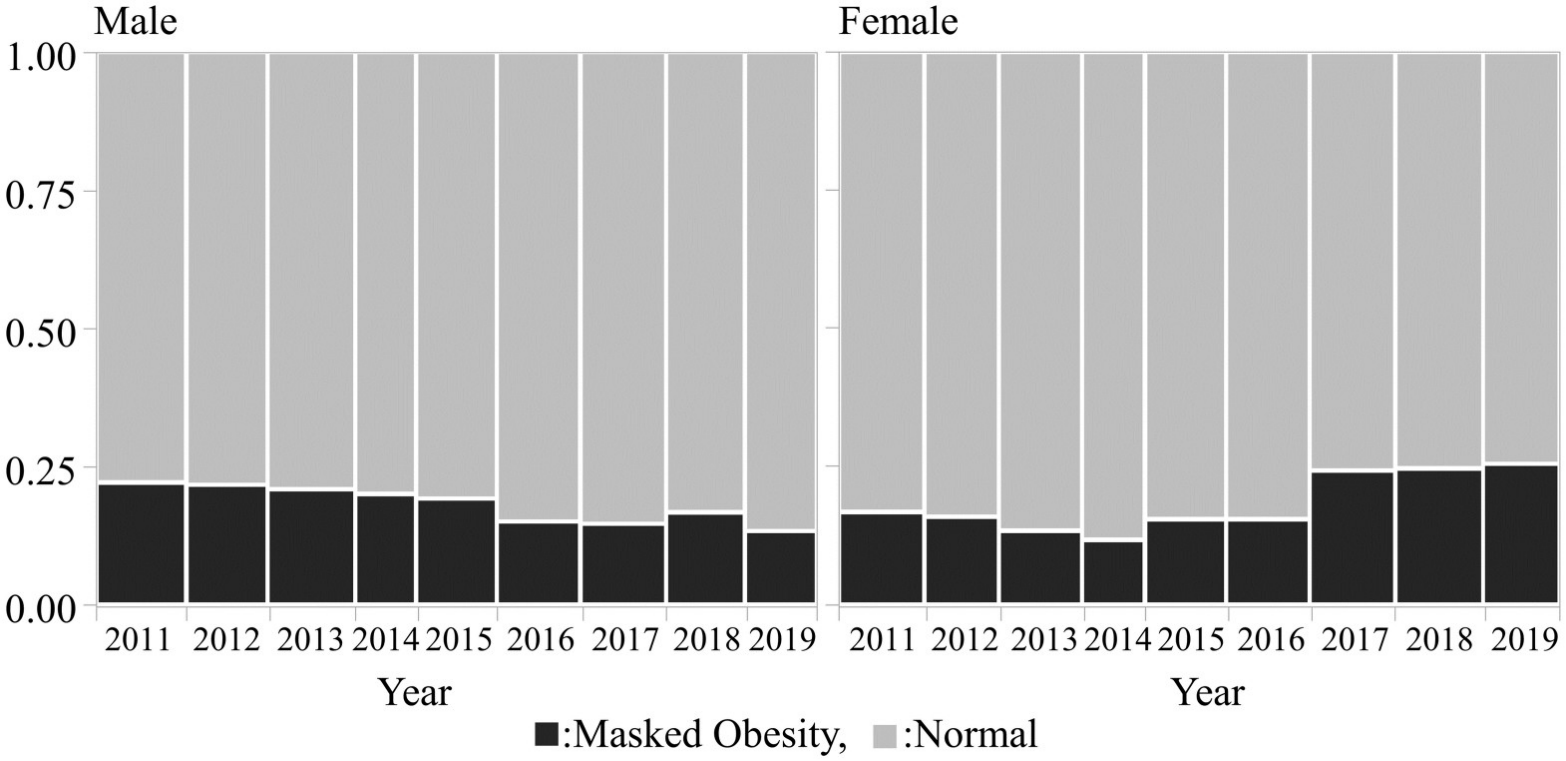
The change over time in the proportion of masked obesity in male and female students between 2011 and 2019.

Fig 1 shows the change over time in the proportion of MO in male and female students between 2011 and 2019. The percentage of male students with MO was 22.2% in 2011 and 13.4% in 2019, showing a significant decrease over time (p < 0.001). The percentage of female students with MO was 17.0% in 2011 and 25.8% in 2019, showing a significant increase over time (p < 0.001).

**Fig 2:**
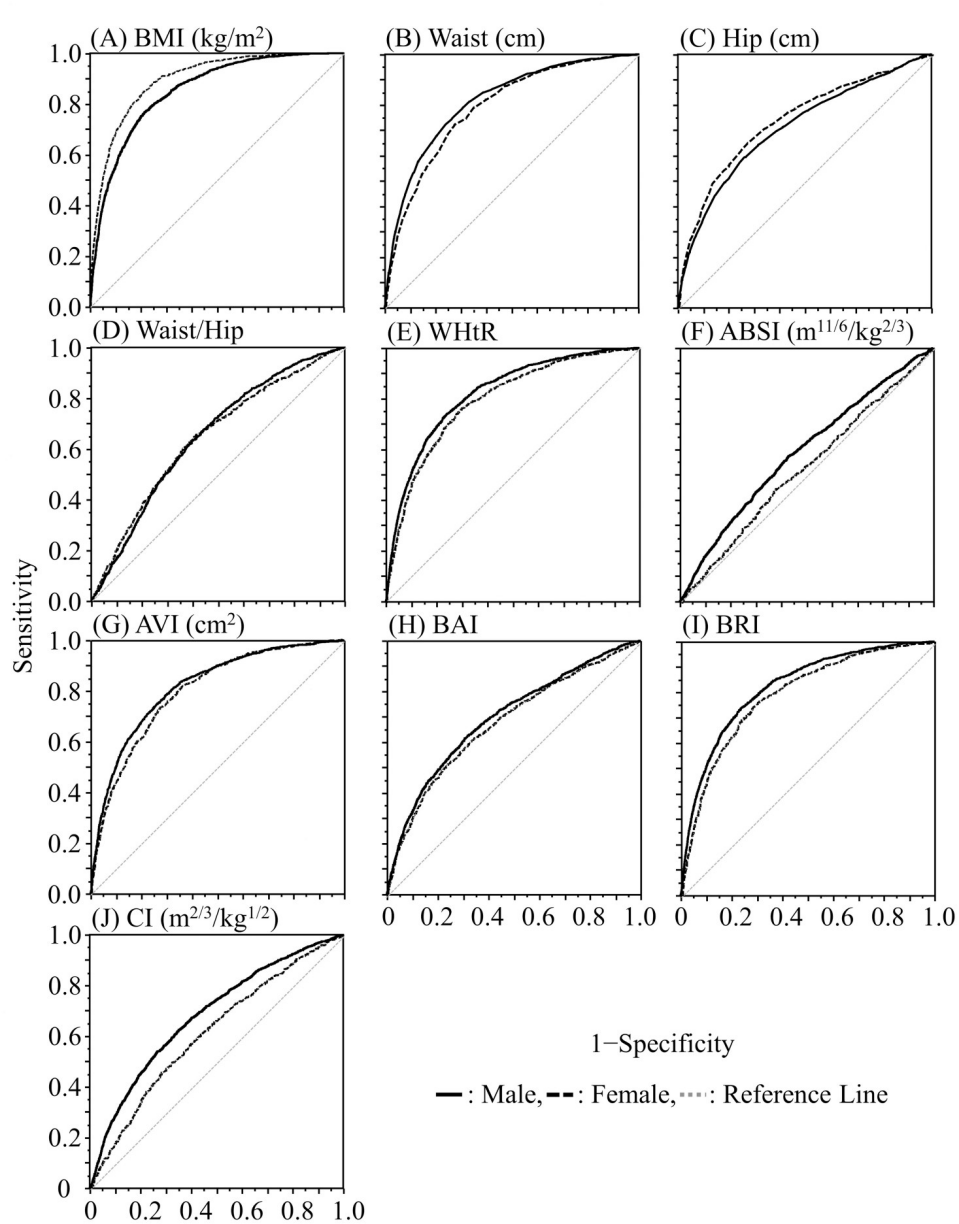
ROC curves for masked obesity of each anthropometric measure and body indices.

Table 1 shows the results of comparison of anthropometric indices in the MO and normal groups. Table 2 and Fig 2 show the ROC curve, AUC, cutoff values, sensitivity, specificity, and odds ratio of anthropometric indices for MO in all participants. Mean BMI values in the MO and normal groups were 23.0 kg/m^2^ and 20.8 kg/m^2^, respectively, in males; and 22.8 kg/m^2^ and 20.4 kg/m^2^, respectively, in females, and significant differences were found for BMI between the MO and normal students (p < 0.001) despite the equal BMI ranges. There was no significant difference in age or height between the MO and normal groups. All other physical measurements were significantly higher in the MO group than in the normal group (p < 0.01 to 0.001). The AUC of BMI was 0.852 for males and 0.898 for females, indicating moderate to high predictive ability for MO. The AUC of WC was 0.815 for males and 0.792 for females, also showing moderate predictive ability for MO, with a cutoff value of 76.6 cm for males and 71.3 cm for females. Regarding body indices, the AUC of AVI showed the highest value, of 0.817 for males and 0.800 for females, with cutoff values of 11.7 for males and 10.4 for females. When each anthropometric index was classified by cut-off value, the odds ratios and 95% confidence intervals (OR [95% CI]) for BMI and WC were 12.09 (10.73–13.63) and 8.29 (7.40–9.28), respectively, for males; and 21.25 (17.50–25.79) and 7.01 (5.97–8.23), respectively, for females. The OR (95% CI) for AVI was 8.61 (7.64–9.71) for males and 7.36 (6.25–8.66) for females.

**Table 1:** Comparison of anthropometric indices of university students with the masked obesity and normal groups.

	Masked obesity	Normal	p-value
Male	(n = 1,868)	(n = 8,300)	
Age (year)	18.5±0.9	18.5±0.8	0.327
Height (cm)	171.6±5.9	171.7±5.7	0.563
Weight (kg)	67.7±6.2	61.5±5.8	< 0.001
%BF (%)	22.3±2.5	15.0±3.0	< 0.001
BMI (kg/m^2^)	23.0±1.3	20.8±1.5	< 0.001
WC (cm)	79.8±5.3	73.7±4.7	< 0.001
HC (cm)	93.0±5.6	88.9±5.7	< 0.001
WHR	0.86±0.06	0.83±0.06	< 0.001
WHtR	0.47±0.03	0.43±0.03	< 0.001
ABSI (m^11/6^/kg^2/3^)	0.075±0.004	0.074±0.004	< 0.001
AVI (cm^2^)	12.94±1.67	11.10±1.37	< 0.001
BAI (%)	23.42±2.66	21.53±2.69	< 0.001
BRI	2.75±0.51	2.15±0.45	< 0.001
CI (m^2/3^/kg^1/2^)	1.17±0.06	1.13±0.05	< 0.001
Female	(n = 915)	(n = 4,039)	
Age (year)	18.5±0.7	18.4±0.7	0.275
Height (cm)	158.3±5.5	158.6±5.4	0.138
Weight (kg)	57.1±5.2	51.4±4.6	< 0.001
%BF (%)	32.4±2.2	24.9±3.2	< 0.001
BMI (kg/m^2^)	22.8±1.3	20.4±1.2	< 0.001
WC (cm)	74.4±5.2	68.8±4.7	< 0.001
HC (cm)	93.7±4.9	89.7±4.6	< 0.001
WHR	0.80±0.06	0.77±0.05	< 0.001
WHtR	0.47±0.03	0.43±0.03	< 0.001
ABSI (m^11/6^/kg^2/3^)	0.074±0.004	0.073±0.004	0.004
AVI (cm^2^)	11.42±1.51	9.84±1.25	< 0.001
BAI (%)	29.10±2.67	26.96±2.60	< 0.001
BRI	2.84±0.59	2.23±0.50	< 0.001
CI (m^2/3^/kg^1/2^)	1.14±0.07	1.11±0.07	< 0.001

Mean ± standard deviation. %BF: body fat percentage, BMI: body mass index, WC: waist circumference, HC: hip circumference, WHR: WC to HC ratio, WHtR: WC to height ratio, ABSI: body shape index, AVI: abdominal volume index, BAI: body adiposity index, BRI: body roundness index, CI: conicity index. p-statistical significance obtained using the Mann–Whitney U test.

**Table 2:** AUC, cut-off value, sensitivity, specificity, and odds ratio of anthropometric indices for masked obesity in all participants.

	AUC	Cut-off value	Sensitivity	Specificity	OR (95% CI)	p-value
Male						
BMI (kg/m^2^)	0.852	22.0	0.769	0.784	12.09 (10.73–13.63)	< 0.001
WC (cm)	0.815	76.6	0.726	0.758	8.29 (7.40–9.28)	< 0.001
HC (cm)	0.710	92.5	0.594	0.739	4.14 (3.73–4.59)	< 0.001
WHR	0.648	0.84	0.635	0.599	2.60 (2.35–2.89)	< 0.001
WHtR	0.821	0.45	0.739	0.761	9.05 (8.06–10.15)	< 0.001
ABSI (m^11/6^/kg^2/3^)	0.587	0.075	0.574	0.572	1.80 (1.63–1.99)	< 0.001
AVI (cm^2^)	0.817	11.7	0.785	0.702	8.61 (7.64–9.71)	< 0.001
BAI (%)	0.698	22.9	0.616	0.693	3.62 (3.26–4.02)	< 0.001
BRI	0.821	2.42	0.739	0.761	9.05 (8.06–10.15)	< 0.001
CI (m^2/3^/kg^1/2^)	0.681	1.15	0.624	0.649	3.07 (2.77–3.41)	< 0.001
Female						
BMI (kg/m^2^)	0.898	21.4	0.843	0.799	21.25 (17.50–25.79)	< 0.001
WC (cm)	0.792	71.3	0.725	0.727	7.01 (5.97–8.23)	< 0.001
HC (cm)	0.743	92.3	0.649	0.731	5.02 (4.31–5.85)	< 0.001
WHR	0.646	0.78	0.607	0.639	2.73 (2.35–3.16)	< 0.001
WHtR	0.796	0.45	0.766	0.700	7.66 (6.48–9.05)	< 0.001
ABSI (m^11/6^/kg^2/3^)	0.531	0.074	0.447	0.627	1.36 (1.18–1.57)	< 0.001
AVI (cm^2^)	0.800	10.4	0.738	0.723	7.36 (6.25–8.66)	< 0.001
BAI (%)	0.723	28.2	0.660	0.683	4.19 (3.60–4.88)	< 0.001
BRI	0.796	2.43	0.766	0.700	7.66 (6.48–9.05)	< 0.001
CI (m^2/3^/kg^1/2^)	0.618	1.14	0.458	0.722	2.19 (1.89–2.54)	< 0.001

%BF: body fat percentage, BMI: body mass index, WC: waist circumference, HC: hip circumference, WHR: WC to HC ratio, WHtR: WC to height ratio, ABSI: body shape index, AVI: abdominal volume index, BAI: body adiposity index, BRI: body roundness index, CI: conicity index, OR: odds ratio, 95% CI: 95% confidence interval. p: statistical significance obtained using univariate logistic regression analysis.

Tables 3 and 4 show the relationship of MO to ideal body weight and lifestyle habits. The percentages of male students in the MO and normal groups were 69.2% and 32.3% in the decreasing group, 23.9% and 47.3% in the maintenance group, and 6.9% and 20.4% in the increasing group, respectively. Multivariate logistic regression analysis showed that MO in male students was significantly associated with BMI (OR: 2.49, 95% CI: 2.37–2.61, p < 0.001) and the desire for weight loss (OR: 1.76, 95% CI: 1.53–2.02, p < 0.001), five macronutrient intakes (OR: 0.79, 95% CI: 0.67–0.93, p = 0.004), rice and wheat intakes (OR: 1.22, 95% CI: 1.01–1.47, p = 0.042), sleep duration of < 7hr (OR: 0.85, 95% CI: 0.74–0.98, p = 0.022), and exercise habit (OR: 0.71, 95% CI: 0.63–0.81, p < 0.001). In contrast, the percentage of female students who had the desire for weight loss was 98.5% in the MO group and 89.6% in the normal group, indicating that the majority of females had the desire for weight loss. Multivariate logistic regression analysis showed that in female students, MO was significantly associated with BMI (OR: 3.59, 95% CI: 3.31–3.90, p < 0.001), balanced diet intake (OR: 0.79, 95% CI: 0.64–0.99, p = 0.044) and exercise habit (OR: 0.65, 95% CI: 0.51–0.82, p < 0.001).

**Table 3:** Relationship between masked obesity and weight control orientation or lifestyle habits in male students.

	Male			
	Masked obesity (%)		aOR (95% CI)	p-value
	Yes	No		
	(n = 1,868)	(n = 8,300)		
BMI (kg/m^2^)			2.49 (2.37–2.61)	<0.001
Ideal body weight				
Decreasing	69.2	32.3	1.76 (1.53–2.02)	<0.001
Increasing	6.9	20.4	0.89 (0.70–1.13)	0.328
Keeping	23.9	47.3	Ref.	Ref.
Breakfast	84.7	85.0	0.98 (0.83–1.17)	0.847
Snack	79.4	83.8	0.90 (0.77–1.05)	0.182
Macronutrient	38.0	42.8	0.79 (0.67–0.93)	0.004
Rice/wheat	69.7	68.3	1.22 (1.01–1.47)	0.042
Meat	66.7	66.6	0.83 (0.67–1.02)	0.081
Fat	63.2	60.1	1.19 (0.98–1.44)	0.080
Vegetable	44.4	47.5	1.02 (0.86–1.19)	0.849
Fruit	29.6	34.0	0.87 (0.74–1.02)	0.077
Balanced diet	48.6	52.3	0.93 (0.80–1.07)	0.298
Sleep duration				
< 7hr	59.7	60.9	0.85 (0.74–0.98)	0.022
≥ 8hr	10.9	10.1	1.06 (0.86–1.32)	0.574
7 ≤, < 8hr	29.3	29.1	Ref.	Ref.
Sleep onset time	56.0	55.6	0.99 (0.87–1.12)	0.828
Exercise habits	37.6	38.4	0.71 (0.63–0.81)	<0.001
Year				
2019	7.2	10.4	0.41 (0.32–0.54)	<0.001
2018	10.1	11.3	0.52 (0.41–0.66)	<0.001
2017	9.5	12.2	0.45 (0.35–0.58)	<0.001
2016	9.6	12.2	0.51 (0.39–0.65)	<0.001
2015	11.7	10.8	0.76 (0.59–0.96)	0.024
2014	9.5	8.5	0.86 (0.67–1.11)	0.257
2013	13.8	11.6	0.90 (0.71–1.14)	0.377
2012	13.4	10.9	0.85 (0.67–1.07)	0.168
2011	15.3	12.1	Ref.	Ref.

aOR: adjusted odds ratio, 95% CI: 95% confidence interval. p: statistical significance obtained using multivariate logistic regression analysis.

**Table 4:** Relationship between masked obesity and weight control orientation or lifestyle habits in female students.

	Female			
	Masked obesity (%)		aOR (95% CI)	p-value
	Yes	No		
	(n = 915)	(n = 4,039)		
BMI (kg/m^2^)			3.59 (3.31–3.90)	< 0.001
Ideal body weight				
Decreasing	98.5	89.6	1.56 (0.85–2.84)	0.150
Increasing	0.0	0.2	N/A	0.994
Keeping	1.5	10.2	Ref.	Ref.
Breakfast	90.6	92.0	0.76 (0.54–1.06)	0.106
Snack	87.5	90.9	0.86 (0.63–1.18)	0.354
Macronutrient	33.1	33.9	0.87 (0.67–1.11)	0.257
Rice/wheat	63.6	62.1	1.18 (0.89–1.56)	0.250
Meat	60.7	57.6	1.13 (0.82–1.54)	0.459
Fat	60.8	57.3	1.06 (0.78–1.44)	0.723
Vegetable	44.0	47.8	0.92 (0.72–1.17)	0.489
Fruit	34.6	35.6	1.08 (0.85–1.37)	0.536
Balanced diet	51.0	52.0	0.79 (0.64–0.99)	0.044
Sleep duration				
< 7hr	70.1	71.7	0.83 (0.66–1.05)	0.122
≥ 8hr	6.9	6.7	0.94 (0.62–1.43)	0.764
7 ≤, < 8hr	23.1	21.6	Ref.	Ref.
Sleep onset time	55.7	50.0	1.21 (1.00–1.47)	0.051
Exercise habits	19.2	21.2	0.65 (0.51–0.82)	< 0.001
Year				
2019	14.5	9.5	2.03 (1.39–2.97)	< 0.001
2018	16.4	11.3	1.85 (1.28–2.68)	< 0.001
2017	15.4	10.9	2.07 (1.42–3.01)	< 0.001
2016	9.9	12.1	1.08 (0.72–1.62)	0.701
2015	9.6	11.7	1.08 (0.72–1.61)	0.717
2014	5.6	9.3	0.86 (0.55–1.35)	0.518
2013	8.5	12.3	0.78 (0.52–1.18)	0.237
2012	8.9	10.6	0.67 (0.44–1.01)	0.054
2011	11.1	12.3	Ref.	Ref.

aOR: adjusted odds ratio, 95% CI: 95% confidence interval. p: statistical significance obtained using multivariate logistic regression analysis.

Table 5 shows the relationship of hypertension to BMI, WC, HC, and MO. In male students, a significant positive association was observed between hypertension and BMI (p < 0.001), WC (p = 0.005), and MO (p = 0.004), with OR (95% CI) of 1.14 (1.08–1.20), 1.02 (1.01–1.04), and 1.29 (1.09–1.53), respectively. In female students, a significant positive association was observed between hypertension and WC (p < 0.001), with OR (95% CI) of 1.06 (1.03–1.10). The cutoff values for BMI and WC were 20.9 kg/m^2^ and 77.0 cm in male students, whereas that for WC in female students was 75.2 cm.

**Table 5:** Relationship of hypertension to body mass index, waist circumference, hip circumference, and masked obesity.

	Univariate		Multivariate		Cutoff value
	OR (95% CI)	p-value	OR (95% CI)	p-value	
Male		< 0.001			
BMI (kg/m^2^)	1.23 (1.19–1.28)	< 0.001	1.14 (1.08–1.20)	< 0.001	20.9
WC (cm)	1.06 (1.05–1.07)	< 0.001	1.02 (1.01–1.04)	0.005	77.0
HC (cm)	1.03 (1.02–1.04)	< 0.001			
MO (%)	1.92 (1.67–2.22)		1.29 (1.09–1.53)	0.004	
Female		0.036			
BMI (kg/m^2^)	1.14 (1.01–1.28)	< 0.001			
WC (cm)	1.06 (1.03–1.10)	0.317	1.06 (1.03–1.10)	<0.001	75.2
HC (cm)	1.02 (0.98–1.06)	0.617			
MO (%)	1.13 (0.70–1.84)				

BMI: body mass index, WC: waist circumference, HC: hip circumference, MO: masked obesity, OR: odds ratio, 95% CI: 95% confidence interval. p: statistical significance obtained using logistic regression analysis. The effectiveness of explanatory variables was evaluated using the stepwise method with a significance level of 0.05 (forward-backward).

## Discussion

MO is defined as normal BMI but with high %BF. Suzuki et al. [25] defined MO as weight of < 120% of the JASSO standard BMI, with %BF of ≥ 25% in males and ≥ 30% in females. Fujise et al. [2] defined MO as a normal BMI based on JASSO and a %BF of ≥ 20% in males and ≥ 30% in females. Based on that study, we defined MO as 18.5 ≤ BMI < 25 kg/m^2^ and %BF of ≥ 20% in males, and %BF of ≥ 30% in females. In the present study, the percentages of male and female students with MO were 18.4% and 18.5%, respectively. In addition, the percentage of male students with MO decreased, whereas that of female students increased over time between 2011 and 2019. The reason for the decrease in the percentage of male students with MO over time remains unclear; however, there is concern that MO could continue to increase in females in the future.

In the present study, the mean BMI values of male and female students with MO were 23.0 kg/m^2^ and 22.8 kg/m^2^, respectively, which is significantly higher than those of the normal groups. Additionally, mean WHR values of the MO and normal groups were 0.86 and 0.83 for male students and 0.80 and 0.77 for female students, respectively, and the mean was significantly higher in the MO groups than in the normal groups. WHR is used as an indicator of obesity, with a cutoff value of ≥ 0.95–1.0 in males and ≥ 0.80–0.85 in females [26]. In the present study, the cutoff values of BMI and WHR for predicting MO were 22.0 kg/m^2^ and 0.84 in males and 21.4 kg/m^2^ and 0.78 in females, respectively, which are lower than those for predicting obesity reported in previous studies [17, 26]. Recently, new anthropometric indices such as ABSI, AVI, BAI, BRI, and CI, which can estimate body fat distribution, have been used to evaluate obesity [7, 8]. However, little is known about the relationship between MO and anthropometric indices among Japanese university students. We compared the AVI and BRI values, which had high predictive ability in our study, with those reported in previous studies for predicting metabolic syndrome [8, 27, 28]. The cutoff values of AVI in previous studies were 14.3–16.2 in males and 12.8–13.0 in females, whereas those of the present study were 11.7 in males and 10.4 in females. In addition, the cutoff value of BRI in previous studies was 3.47–3.94 in males and 3.43–3.77 in females, whereas that of our study was 2.42 in males and 2.43 in females. The cutoff values of AVI and BRI were lower in the present study than those in previous studies, which suggests that new cutoff values should be set for determining MO using anthropometric indices, and that the criteria should include sex and age.

The percentage of the present female students with the desire for weight loss was 98.5% in the MO group and 89.6% in the normal group. Japanese females have a strong desire for weight loss, as reported in a study of ideal body image that found more than 90% of female university students “want to be thin” [29]. More than 70% of female university students have experienced dieting behaviors [29]. It has been suggested that these desires and behaviors are risk factors for MO [2, 4]. Thus, intervention for appropriate assessment of body shape is needed in Japanese university students. Meanwhile, the percentage of the present male students with the desire for weight loss was higher in the MO group than in the normal group. Kagawa et al. [30] suggested that Japanese males are more likely to overestimate their own weight than are western males. Although there are limited data on a desire for weight loss among males, such a desire may induce inappropriate dieting behaviors that are risk factors for MO.

The MO groups in the present study tended to have low intake of the five macronutrients (in males) and low intake of a balanced diet (in females). A previous study found that young Japanese females with MO had low intake of vegetables and beans [4], in agreement with the present results. Further research is needed to identify the exact amounts of intake for each nutrient, which was not possible in the present study as the survey method was based on a self-rating of lifestyle habits. According to the National Health and Nutrition Survey [18], the frequency of intake of a balanced diet is lower among Japanese aged 20–29 years than in other generations. It is also a concern that the easy availability of food at fast food restaurants, convenience stores, and vending machines has led to disordered eating habits among young people [31]. Intake of a well-balanced diet is important, with each nutrient consumed without excess or deficiency.

The percentage of male students with sleep duration of < 7 hours was lower in the MO group than in the normal group. The percentage of those who average 7–8 hours of sleep per day is highest among Japanese aged 20–29 years, but the percentage of those who feel sleepy during the daytime is high [18]. This is due to the pre-bedtime use of cell phones and video games, which can interfere with sleep quality. Furthermore, it has been reported that the prevalence of hypertension and metabolic syndrome, which are risk factors for cardiovascular disease, was higher in those with sleep duration of < 7 or ≥ 8 hours compared with those with sleep duration of 7–8 hours, and the relationship between sleep duration and prevalence showed a U shape [32, 33]. Although the percentage of MO was lower in those with sleep duration of < 7 hours, the results of previous studies suggest that it is important to ensure 7–8 hours of sleep.

The present study found that compared with the normal group, fewer male and female students in the MO groups were in the habit of exercising. Another study has reported that young Japanese females with MO had no exercise habits [4]. MO can be induced by a decrease in muscle mass and bone mass caused by a lack of exercise [2]. Meanwhile, data from the National Health and Nutrition Survey [18] reveal that the percentage of Japanese aged 20–29 years with exercise habits is 28.4% in males and 12.9% in females, with no significant fluctuation over time in males but a significant decrease in females over the past decade. Regarding the desire to improve their exercise habits, data from the National Health and Nutrition Survey [18] reveal the percentage of respondents who answered “interested but have no orientation to improve” was high among males and females aged 20–29 years, and was particularly high among those who had no exercise habits. Therefore, how exercise habits can be established is an issue to be addressed in the future.

In the present study, hypertension was significantly associated with BMI, WC, and MO in male students, and with WC in female students. Uchiyama et al. [34] found that hypertension in Japanese university students was associated with higher BMI, and the prevalence increased as BMI increased. In a previous study that defined MO as weight of < 120% of the JASSO standard BMI, with %BF of ≥ 25% in males and ≥ 30% in females, SBP and DBP values in participants who underwent health screening were higher in the MO group than the normal group [25]. In the present study, however, we did not observe a significant relationship between hypertension and MO in female students, possibly because of the small percentage of female students with hypertension. These findings suggest that MO may be a risk factor for hypertension as well as obesity. We additionally observed a significant positive relationship between hypertension and WC in both male and female students in our study. The diagnostic criteria for metabolic syndrome are established as WC of ≥ 85 cm in males and ≥ 90 cm in females [35]. However, our results showed that mean WC was within these limits even in the MO groups. In a previous study of university students [36], the mean value of WC was 73.3 cm in males and 67.8 cm in females, which was lower than the diagnostic criteria. As WC has been shown to increase with aging [18], management of body shape is needed from young adulthood.

This study has several research limitations. First, it was designed as a cross-sectional survey, which cannot provide a causal inference or demonstrate a mechanistic connection between MO and lifestyle habits. Second, the study was conducted using a self-administered questionnaire, which may not accurately assess lifestyle habits. Third, regarding dietary habits, as we did not assess intake of nutrients in detail, caution should be exercised in interpretation. However, this is the first study to investigate the relationship between MO and lifestyle habits and the effects of MO on health among Japanese university students. The present results provide new evidence on MO management in university students.

## Conclusion

The percentages of students with MO in 2019 were 13.4% for males with a decreasing trend, and 25.8% for females with an increasing trend. MO was negatively associated with the intake of five macronutrients, balanced diet intake, or exercise habits. Hypertension was significantly associated with BMI, WC, and MO in males, and with WC in females. These findings suggest that intervention for MO is needed in female university students, which could be achieved by adjusting their eating and exercise habits. Furthermore, as MO in young people may be a risk factor for hypertension, intervention from a young age is important.

## Supporting information

S1 Table Questionnaire entitled survey on lifestyle habits. (DOCX)
