# Supplementary material for: Relationship of masked obesity to self-reported lifestyle habits, ideal body image, and anthropometric measures in Japanese university students: A cross-sectional study
Source: PLoS One. 2023 Feb 21;18(2):e0281599. doi: 10.1371/journal.pone.0281599 (PMC9943004; doi:10.1371/journal.pone.0281599)
Supplement: S1 Table — (DOCX) [file pone.0281599.s001.docx]

**Supporting information**

| **S1 Table. Questionnaire entitled survey on lifestyle habits.** | |
| --- | --- |
| 1. Age | year |
| 2. Sex | □ Male □ Female |
| 3. Ideal body weight | □ Decreasing □ Maintenance □ Increasing |
| Eating habits | |
| 4. Meal frequency | □ No □ 1 time □ 2 times □ 3 times □ 4 times  (□ Breakfast □ Lunch □ Dinner □ Midnight snack) |
| 5. Snack | □ Daily □ Occasionally □ I used to snack □ I do not snack |
| 6. Select 5 or 6 normally |  |
| Macronutrient | Unsatisfactory 1 2 3 4 5 6 7 8 9 10 Satisfactory |
| Rice/wheat | Unsatisfactory 1 2 3 4 5 6 7 8 9 10 Satisfactory |
| Meat | Unsatisfactory 1 2 3 4 5 6 7 8 9 10 Satisfactory |
| Fat | Unsatisfactory 1 2 3 4 5 6 7 8 9 10 Satisfactory |
| Vegetable | Unsatisfactory 1 2 3 4 5 6 7 8 9 10 Satisfactory |
| Fruit | Unsatisfactory 1 2 3 4 5 6 7 8 9 10 Satisfactory |
| Balanced diet | Unsatisfactory 1 2 3 4 5 6 7 8 9 10 Satisfactory |
| Sleeping habits | |
| 7. Sleep duration | hours |
| 8. Sleep onset time | minutes |
| Exercise habits | |
| 9. Exercise frequency | times/week |
| 10. Exercise duration | min/time |
